# Distinct Functional Signatures of Human Olfactory and Respiratory Mucus Revealed by Proteomics Combined With Machine Learning

**DOI:** 10.1002/prca.70049

**Published:** 2026-07-17

**Authors:** Romain Topalian, Anna Kristina Hernandez, Karoline Lantzsch, Philipp Hubel, Chrystelle Mavoungou, Frank Rosenau, Jens Pfannstiel, Thomas Hummel, Katharina Schindowski

**Affiliations:** ^1^ Institute of Pharmaceutical Biotechnology Ulm University Ulm Germany; ^2^ Institute For Applied Biotechnology Biberach University of Applied Sciences Biberach Germany; ^3^ Smell & Taste Clinic Department of Otorhinolaryngology Faculty of Medicine Carl Gustav Carus Technische Universität Dresden Dresden Germany; ^4^ Department of Otolaryngology – Head and Neck Surgery Philippine General Hospital University of the Philippines – Manila Manila Philippines; ^5^ Department of Otolaryngology – Head and Neck Surgery Asian Hospital and Medical Center Muntinlupa Philippines; ^6^ Core Facility Hohenheim Mass Spectrometry Unit University of Hohenheim Stuttgart Germany

**Keywords:** human nasal olfactory and respiratory mucus, PLS‐DA, proteomics, Reactome database, secretome

## Abstract

**Background:**

The nasal cavity includestwo distinct epithelial regions: olfactory and respiratory which fulfilldifferent roles. Despite their differences, their mucus composition, however, is yet not well elucidated.

**Methods:**

To analyze themucosal secretome, samples from the human olfactory mucus (OM) and respiratorymucus (RM) were collected in a volunteer study with 25 normosmic individualsand analyzed using label‐free quantitative proteomics (LFQ), supervised machinelearning (Partial Least Squares Discriminant Analysis, PLS‐DA), functionalenrichment via Gene Ontology (GO) and pathway analyses at Reactome database.

**Results:**

A total of 1,780high‐confidence proteins were quantified across 50 samples. The optimizedPLS‐DA model achieved robust discrimination between OM and RM (AUC = 0.93 ±0.04), identifying distinct molecular signatures. Cross‐validation across GO,Reactome, and PLS‐DA macro‐category analyses confirmed the robustness andbiological coherence of these findings.

**Conclusions:**

Overall, this study defines twocomplementary mucosal ecosystems: a dynamic olfactory mucus optimized for highmitochondrial activity, (non‐motile) ciliary renewal, and autophagy, and animmune‐active respiratory mucus specialized in host defense, providing acomprehensive molecular framework of nasal regional specialization.

**Abbreviations**
FDR: False Discovery Rate
GO: Gene Ontology
iBAQ: intensity‐Based Absolute Quantification
LFQ: Label‐Free Quantification
OM: Olfactory Mucus
PLS‐DA: Partial Least Squares Discriminant Analysis
RM: Respiratory Mucus
SVD: Singular Value Decomposition
PCA: Principal Component Analysis
UMAP: Uniform Manifold Approximation and Projection

**Statement of Significance of the Study**

This proteomic investigation of human olfactory and respiratory mucus represents a significant advance, providing a direct molecular snapshot of the airway surface in human volunteers: a context that is uniquely relevant to clinical and translational research. Unlike the many studies of general nasal secretions, our study specifically isolated and compared mucus from both the olfactory cleft and the respiratory nasal mucosa, thereby offering an unprecedented comparison of niche‐specific proteomes in vivo. Given the relative scarcity of published human datasets focused on olfactory‐cleft mucus, this work not only fills a critical gap but also opens pathways to identify biomarkers of mucosal immunity, odorant metabolism and mucosal barrier function that are directly applicable to human physiology and disease. Furthermore, this study provides the first integrative proteomic comparison of human olfactory and respiratory mucus using label‐free mass spectrometry combined with supervised machine learning. These findings define molecular benchmarks for nasal regional specialization and offer valuable insight for future research on nasal pathophysiology and intranasal drug delivery strategies.

## 1 Introduction

Nasal mucus serves as a multifunctional barrier that traps airborne particles and pathogens, protecting underlying epithelia [1, 2, 3, 4]. Beyond this mechanical defense, it contributes to mucosal immunity and olfaction, reflecting regional specialization within the nasal cavity [5, 6, 7].

The nasal cavity harbors two distinct epithelial areas: the olfactory epithelium and the respiratory epithelium [8, 9]. Although adjacent, they differ markedly in cellular composition and physiological. The olfactory epithelium is specialized for the initial peripheral processing of odorant information and is characterized by the presence of olfactory sensory neurons, supporting cells, and secretory elements that produce odorant‐binding proteins and lipocalins, which facilitate odorant transport and recognition [10, 11, 12]. In contrast, the respiratory epithelium consists predominantly of motile ciliated and goblet cells responsible for mucus secretion and mucociliary clearance resulting in innate immune defense through the secretion of mucins and secreted antimicrobial proteins [13, 14]. Although adjacent, they differ markedly in cellular composition and physiological roles. Previous studies have characterized the olfactory mucus (OM) proteome [15], but a direct comparison with respiratory mucus (RM) has yet not been systematically performed.

Nasal mucus is a complex fluid containing secreted, enzymatic, and immune‐related proteins that reflect epithelial physiology and environmental exposure [16, 17]. Advances in mass spectrometry–based proteomics [18, 19] has enabled the unbiased and quantitative characterization of the molecular composition of nasal mucus [20, 21], providing insight into regional and functional specialization within the nasal cavity [15, 22, 23, 24]. Previous proteomic studies have demonstrated regional differences in mucin expression, particularly MUC5AC and MUC5B, reflecting the distinct secretory functions of the olfactory and respiratory epithelia [10, 12, 13, 25, 26, 27]. Other studies have identified age‐, sex‐, and disease‐related variations in the nasal mucus proteome, highlighting its sensitivity to physiological and pathological changes [15, 20, 28].

Some studies have focused on the OM or RM proteomes using supervised analytical approaches [13, 15, 16, 21, 22, 25, 27, 29], but without comparison. Existing datasets describe compositional differences without linking them to functional specialization [10, 17, 20] and data‐driven models capable of classifying mucus origin or predicting physiological role function are still lacking [23, 30]. Consequently, intra‐individual comparison of the mucus from both nasal regions associated with distinct biological pathway characteristics based on their protein profiles is still lacking.

To address these gaps, this study aims to quantitatively compare the proteomic composition of human olfactory and respiratory nasal mucus using label‐free mass spectrometry, including both label‐free quantification (LFQ) and intensity‐based absolute quantification (iBAQ) [19, 23, 31] and applies supervised machine learning, specifically Partial Least Squares Discriminant Analysis (PLS‐DA), to classify the two mucus types and identify discriminant proteins [30].

Integrating quantitative proteomics, machine learning, and multi‐level pathway enrichment provides a novel framework for understanding the regional specialization of the nasal cavity. This allows the identification of molecular patterns that differentiate OM from RM through large‐scale protein quantification, supervised modeling, and pathway‐level interpretation using GO and Reactome analyses [32, 33]. Such models could provide valuable insights into the molecular mechanisms underlying regional differentiation within the nasal cavity and establish a foundation for future diagnostic or therapeutic applications [18, 28, 34].

## 2 Materials and Methods

### 2.1 Participants, Sample Collection and Preparation

The Ethics Commission of TU Dresden (EK 3620822016) approved this cross‐sectional study, which was conducted in accordance with the principles of the Declaration of Helsinki. Written informed consent was obtained from all participants.

Healthy adults (≥18 years) without olfaction‐related complaints were included. Mucus samples were collected using small cotton and viscose strips (2 × 1 cm, Neurosorb, Vostra, Aachen, Germany), which were inserted into the nasal cavity with endoscopic guidance (Rigid Nasal Endoscope, Karl Storz, Tuttlingen, Germany) over two sites: at the medial aspect of the inferior turbinate (Respiratory Mucosa, RM), and at the olfactory cleft (OC). In order to absorb mucus, these strips remained in place for 5 min before they were removed and placed in micro tubes (2 mL, Sarstedt AG & Co., Nümbrecht, Germany). Prior to further transport, the samples were maintained at 4°C in a refrigerator. Pseudonymized mucus sample pairs were processed at Biberach University of Applied Sciences and Hohenheim University. No samples were containing visible blood contaminations. The study included 25 healthy normosmic participants, as confirmed by the Sniffin’ Sticks Identification Test. The cohort consisted of 12 women and 13 men, with a mean age of 24.80 ± 4.16 years. For each participant, paired OM and RM samples were collected.

### 2.2 Protein Extraction and In‐Gel Digestion

Proteins were solubilized and washed out of the Neurosorb strips with a 1:5 diluted SDS‐sample buffer (0.12% SDS, 2% Glycerol, 0.2% 2‐Mercaptoethanol, 0.0032% Bromophenol Blue, 15 mM Tris‐HCl pH 6.8). Subsequently, the sample buffer was concentrated in a vacuum concentrator (Eppendorf SE, Hamburg, Germany) and loaded loaded onto a 8% acrylamide gel for a short, 10–15 min, SDS–polyacrylamide gel electrophoresis (SDS‐PAGE). Coomassie staining was performed, and the stained regions were cut out of the gel. Subsequently, samples were digested in‐gel with trypsin (Roche, Penzberg, Germany) according to the method of Shevchenko et al. [35]. Peptide concentrations were estimated using a NanoPhotometer (Implen, Model NP80, Munich, Germany).

### 2.3 NanoLC‐MS/MS Analysis

NanoLC‐ESI‐MS/MS experiments were performed on an Ultimate 3000 nano‐RSLC (Thermo Fisher Scientific, Waltham, MA, USA) coupled to an Exploris 480 mass spectrometer (Thermo Fisher Scientific, Waltham, MA, USA) using a Nanospray‐Flex ion source (Thermo Fisher Scientific, Waltham, MA, USA). Peptides were concentrated and desalted on a trap column (5 mm × 30 µm, Thermo Fisher Scientific, Waltham, MA, USA) and separated on a 25 cm × 75 µm nanoEase MZ HSS T3 reversed phase column (100 Å pore size, 1.8 µm particle size; Waters, Milford, CT, USA) operated at constant temperature of 35°C.

Peptides were separated at a flow rate of 300 nL/min using a 150 min method with the following profile: 2%–11% solvent B in 3 min, 11%–30% solvent B in 92 min, 30%–45% solvent B in 30 min, 45%–95% solvent B in 8 min, isocratic at 95% solvent B for 5 min, 95%–2% solvent B in 4 min and isocratic at 2% solvent B for 8 min. Solvents used were 0.1% formic acid (solvent A) and 0.1% formic acid in acetonitrile/H2O (80/20, v/v, solvent B).

Data acquisition was conducted in data‐dependent acquisition (DDA) mode. MS spectra (m/z = 300–1400) were detected in the Orbitrap at a resolution of 60,000 (m/z = 200) using a maximum injection time of 50 ms and an automatic gain control value of 3 × 10^6^. Internal calibration of the Orbitrap analyzer was performed using lock‐mass ions from ambient air as described in Olsen et al. [36]. MS/MS spectra of the top 25 peptide precursors per cycle were generated in the Orbitrap using high energy collision dissociation fragmentation at a resolution of 15,000 and a normalized collision energy of 30%. Further settings for MS/MS spectra included an isolation width of 1.6 Da, a maximum injection time of 50 ms and an automatic gain control value of 8 × 10^4^.

### 2.4 MS Data Analysis and Protein Quantification

Raw files were imported into MaxQuant version 2.0.1.0 for protein identification and LFQ of proteins [37]. Protein identification in MaxQuant was performed using the database search engine Andromeda [38]. MS spectra and MS/MS spectra were searched against Homo sapiens protein sequence database downloaded from [39]. Reversed sequences as decoy database and common contaminant sequences were added automatically by MaxQuant. Mass tolerances of 4.5 ppm (parts per million) for MS spectra and 20 ppm for MS/MS spectra were used. Trypsin was specified as an enzyme, and two missed cleavages were allowed. Carbamidomethylation of cysteines was set as a fixed modification and protein N‐terminal acetylation and oxidation were allowed as variable modifications. The ‘match between runs’ feature of MaxQuant was enabled with a match time window of 0.7 min and an alignment time window of 20 min. Peptide false discovery rate (FDR) and protein FDR thresholds were set to 0.01. Relative quantification was performed by using the iBAQ/LFQ option implemented in MaxQuant.

### 2.5 Label‐Free Quantification (LFQ) and Data Filtering/Preparation

To ensure data quality and reliability, protein groups in LFQ dataset [40, 41] with more than 30% missing values across all samples were excluded from further analysis. For the remaining entries, several imputation strategies were evaluated to estimate missing values and minimize bias. Methods tested included random sampling from a left‐shifted normal distribution, K‐nearest neighbors, and singular value decomposition (SVD) [42, 43].

Following SVD imputation, log_2_ transformation was applied to stabilize variance and approximate a normal distribution of intensity values. Data matrices were then median‐centered within each sample to reduce inter‐sample variability and subsequently z‐score normalized across all samples to standardize the input features prior to statistical and machine learning analyses [44, 45].

### 2.6 Machine Learning Models

Model training was performed using the filtered and imputed LFQ dataset containing paired OM and RM samples. Each sample was represented by a vector of normalized protein intensities (z‐scored log_2_‐transformed LFQ values). Supervised machine learning was applied to classify OM and RM based on their proteomic profiles [34], addressing the high dimensionality and multicollinearity typical of proteomics data [18, 23]. Data were split into 70 % training (*n* = 35) and 30% test sets (*n* = 15).

Both linear models (Logistic Regression, PLS‐DA) and non‐linear algorithms (Extreme Gradient Boosting, fully connected Neural Network, and Convolutional Neural Network) were evaluated [23, 46, 47, 48, 49]. Bayesian optimization was used for hyperparameter tuning, and performance was assessed by five‐fold stratified cross‐validation, ROC curves, and AUC values. Model significance was confirmed by 1000‐fold permutation testing (*p* < 0.001) [50, 51].

Feature importance was extracted from regression coefficients (linear models) or SHapley Additive exPlanations values to quantify the contribution of each protein to class discrimination [52]. These metrics were subsequently integrated into the functional annotation workflow for biological interpretation [53].

### 2.7 Functional Annotation and GO Mapping

Proteins were annotated using gene ontology (GO) terms across the three main categories: Biological Process, Cellular Component, and Molecular Function, taken from the UniProtKB database.

For higher‐level interpretation, related GO terms were manually grouped into broader functional macro‐categories. The relative contribution of each category was calculated from the absolute PLS or machine learning model coefficients, normalized to highlight dominant biological processes distinguishing OM and RM proteomes [40, 54].

### 2.8 Reactome Pathway Enrichment Analysis

Reactome pathway enrichment analysis was performed to complement GO–based annotation. Protein identifiers (UniProt accessions) from the filtered and normalized dataset were uploaded into the Enrichr platform (Reactome 2022, *Homo sapiens* database) [18, 28, 54, 55]. The analysis was conducted using the combined enrichment score that integrates the significance of the overlap (Fisher's exact test) with the magnitude of enrichment (z‐score) [54].

To investigate both global and domain‐specific trends, Reactome data enrichment was computed for the complete list of quantified proteins weighted by their normalized PLS‐DA coefficients, and separately within each of the major biological macro‐categories previously derived from GO Biological Process terms.

### 2.9 Statistical Analysis

All analyses were performed in Python 3.10 (Jupyter Notebook) using *pandas*, *numpy*, *scikit‐learn*, *matplotlib*, and *seaborn*. Two‐sided tests were applied with *p* ≤ 0.05 considered significant.

All scripts were version‐controlled and reproducible across standardized computational environments.

## 3 Results

### 3.1 Dataset Overview and Preprocessing

A total of 50 human nasal mucus samples were analyzed, including 25 OM specimens and 25 RM specimens. Following nanoLC–MS/MS acquisition and data analysis using MaxQuant, 3064 protein groups were initially identified across all samples with at least one unique peptide and a protein FDR of ≤0.01. After applying quality filters to remove proteins with more than 30% missing values, 1780 high‐confidence protein groups remained for quantitative and comparative analyses.

Before data imputation, approximately 28% of total intensity values were missing, a range typical for clinical proteomic samples of high biological complexity [42]. To optimize the reconstruction of missing data while minimizing bias and preserving variance, 12 imputation methods were tested. SVD‐like approach achieved the lowest reconstruction error (RMSE = 0.87, MAE = 0.62), outperforming all other tested algorithms (see Table S1). This method was therefore selected as the final imputation strategy for downstream analyses.

Comparison of intensity histograms before and after imputation confirmed the preservation of data symmetry and variance structure (see Figure S1). The overlap between the two histograms demonstrates that imputation primarily affected low‐intensity missing values without introducing systematic shifts in abundance. Overall, these preprocessing and normalization steps provided a statistically reliable foundation for downstream differential, functional, and machine learning analyses of the OM and RM proteomes.

As an exploratory assessment of potential demographic effects, we evaluated whether sex contributed to the global structure of the proteomic dataset. Principal component analysis (PCA) and uniform manifold approximation and projection (UMAP) visualizations did not reveal clear sex‐driven clustering in the full dataset or within OM and RM samples separately. In addition, exploratory PLS‐DA using sex as the classification label showed weak and unstable discrimination, with a mean cross‐validated AUC of 0.59 ± 0.19 and a mean accuracy of 0.56 ± 0.15. These findings suggest that sex was not a dominant source of proteomic variation in this cohort. Therefore, the subsequent analyses focused on the primary comparison between OM and RM.

### 3.2 Differential Analysis and 1D/2D Annotation Enrichment

**FIGURE 1:**
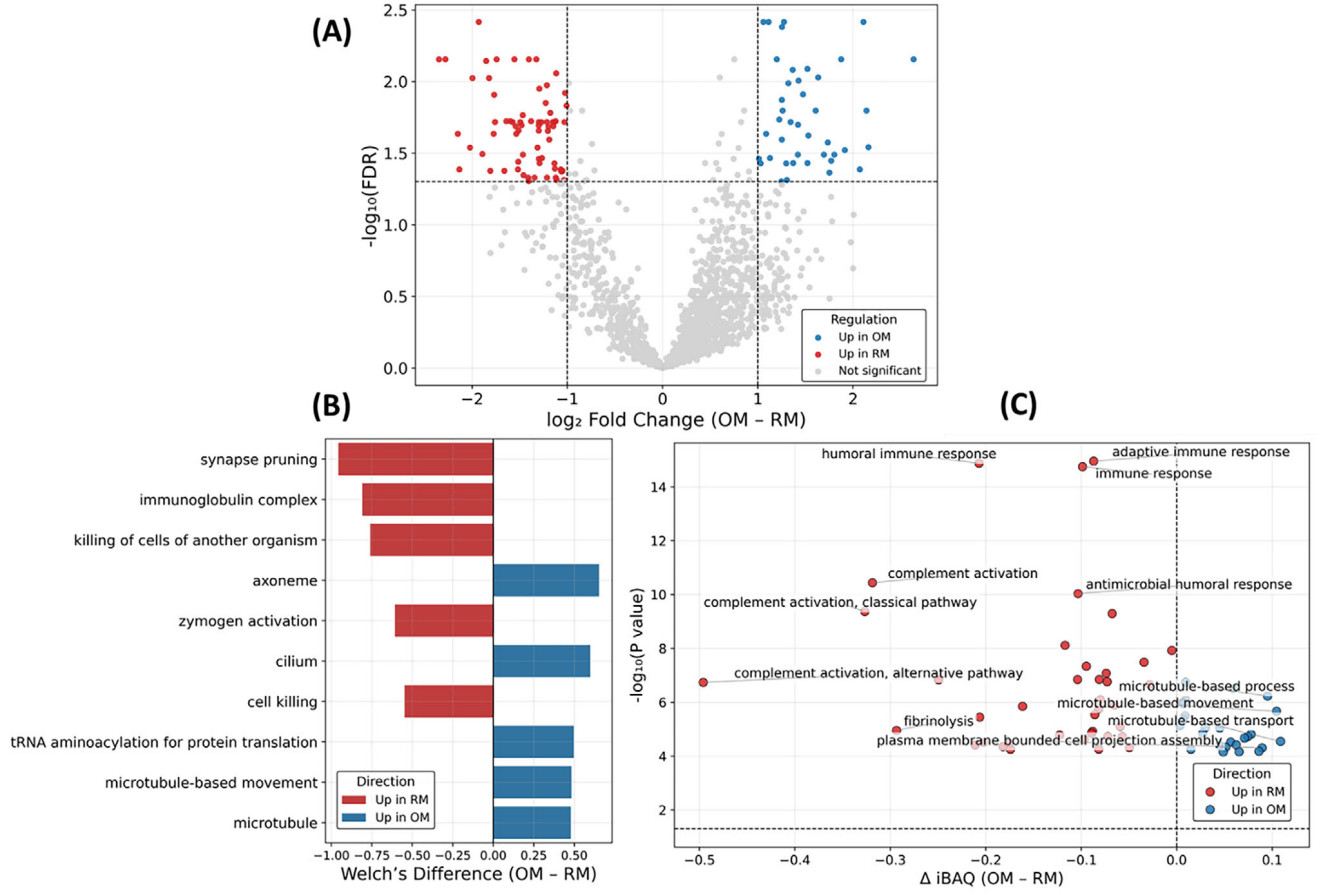
(A) Volcano plot showing the differential abundance of proteins between nasal OM and RM. Each dot represents a quantified protein, plotted according to its log_2_ fold change (OM vs. RM) on the x‐axis and its statistical significance (–log_10_ FDR) on the y‐axis. Proteins significantly enriched in **OM** (log_2_ FC > 1, FDR < 0.05) are shown in **blue**, whereas those enriched in **RM** (log_2_ FC < –1, FDR < 0.05) are shown in **red**. Non‐significant proteins are depicted in **gray**. Dashed lines indicate the applied thresholds for fold‐change and significance (|log_2_ FC| = 1 and FDR = 0.05). (B) One‐dimensional GO enrichment analysis comparing nasal OM and RM. Each bar represents a significantly enriched GO Slim term (FDR < 0.05), derived from Welch's test statistics. Positive enrichment scores (blue bars) indicate functions overrepresented in OM, while negative scores (red bars) correspond to terms enriched in RM. (C) Two‐ GO enrichment analysis comparing nasal OM and RM based on iBAQ‐derived protein abundances. Each point represents a GO Biological Process term, positioned according to the mean difference in iBAQ intensity between OM and RM samples (Δ iBAQ, x‐axis) and the statistical significance of this difference (–log_10_ p, y‐axis). Blue points denote GO terms enriched in OM, whereas red points correspond to terms enriched in **RM** (FDR < 0.05). Dashed lines indicate the thresholds for significance (*p* = 0.05) and neutral abundance difference (Δ iBAQ = 0).

To identify proteomic differences between OM and RM, normalized LFQ intensities were compared using Welch's t‐test with Benjamini–Hochberg correction (*q* < 0.05). The resulting volcano plot (Figure 1A) shows that most proteins clustered around a log_2_ fold change of 0, indicating a largely conserved proteome between both mucus types. However, several proteins exhibited significant directional changes, with higher abundance in OM (blue) or RM (red), revealing region‐specific molecular differences despite global similarity.

To gain functional insight, GO enrichment analyses were performed using one‐dimensional (1D) on LFQ values and two‐dimensional (2D) annotation enrichment on iBAQ values.

The 1D analysis, based on Welch's test statistics, identified overrepresented GO Slim terms (FDR < 0.05) within each mucus type (Figure 1B and Table S2). Distinct functional patterns emerged between the two mucus types. Proteins more abundant in OM were linked to ciliary structure and motility (axoneme, cilium, microtubule‐based movement) and tRNA aminoacylation, indicating enhanced protein translation and mucociliary functionality. In contrast, proteins enriched in RM were primarily associated with synapse pruning, immunoglobulin complex, and protein synthesis‐related functions, suggesting a higher representation of processes linked to molecular recognition. These results show that while both mucus types share a largely overlapping proteome, their associated functions diverge. OM is enriched in structural and mechanical processes whereas RM displays a metabolic (Table S2) and immune orientation.

The 2D annotation enrichment integrated both direction and magnitude of protein abundance differences, visualizing coordinated shifts in biological processes (Figure 1C). Processes related to ciliary and microtubule‐based movement, transport, and structural organization were significantly enriched in OM. In contrast, immune response, complement activation, and antimicrobial humoral defense dominated in RM, indicating enhanced local immune surveillance and barrier protection. These results reinforce the notion that both mucus types contribute to host defense through complementary mechanisms: OM through mechanical‐structural activity and RM through immune and chemical defense processes.

Together, the 1D and 2D GO enrichment analyses revealed distinct yet complementary biological profiles. OM was characterized by structural and motility‐related processes consistent with its mucociliary function. In contrast, RM showed predominant enrichment in immune and antimicrobial pathways, reflecting humoral and chemical protection. These findings highlight that both mucus types contribute to host protection through different strategies: mechanical‐structural in OM and chemical‐immunological in RM. This shows the functional specialization of the nasal environment.

### 3.3 Supervised Classification of OM and RM

Yet, enrichment analyses rely on aggregated statistics and cannot capture inter‐individual variability or complex multivariate dependencies between proteins. To address these limitations, we next applied supervised classification models to identify discriminant protein features driving regional separation.

To assess whether OM and RM samples could be accurately distinguished based on their proteomic signatures, several supervised classification algorithms were implemented and compared.

Among all tested algorithms, the PLS‐DA and XGBoost models achieved the best balance between interpretability and predictive accuracy. The PLS‐DA model reached an average AUC of 0.93 ± 0.04 and an accuracy of 88%. XGBoost reached slightly higher AUC values (0.95 ± 0.03) but displayed signs of overfitting and reduced interpretability. The Logistic Regression and neural networks models also exhibited overfitting, with high training accuracy but unstable validation performance, indicating limited robustness given the dataset size.

**FIGURE 2:**
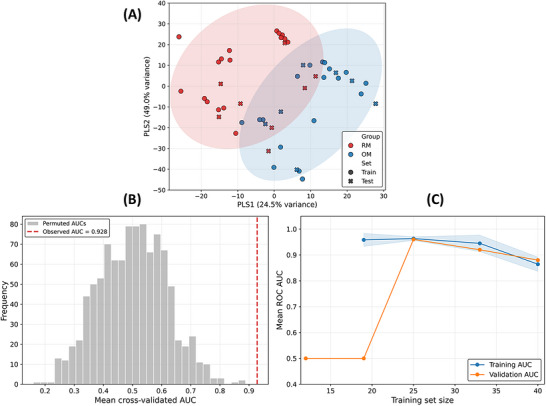
(A) PLS‐DA scores plot of OM and RM samples. Projection of the 50 nasal mucus samples onto the first two latent components (PLS1 and PLS2) obtained from the optimized PLS‐DA model trained on normalized and SVD‐imputed LFQ intensities. Each point represents one sample, with blue and red indicating OM and RM, respectively. Crosses denote test‐set samples, while circles correspond to training samples. The two latent components PLS1 and PLS2 explain the total model variance. Ellipses represent the 95% confidence regions for each group. Model evaluation was performed by five‐fold cross‐validation, with 70% of the samples (*n* = 35) used for training and 30% (*n* = 15) for testing in each fold. (B) Permutation test validating the robustness of the PLS‐DA model. Distribution of mean cross‐validated AUC values obtained from 1000 randomized class‐label permutations (gray bars). The red dashed line indicates the observed mean AUC (0.928) achieved by the optimized PLS‐DA model using the true class labels. (C) Learning curve of the optimized PLS‐DA model. Evolution of the training (blue) and validation (orange) AUC values as a function of the training set size.

The optimized PLS‐DA model, comprising two latent components explaining ∼75 % of total variance, provided the best compromise between predictive power and interpretability. PLS1 captured the main variance between OM and RM, reflecting tissue‐specific proteomic differences, whereas PLS2 represented inter‐individual variability. The resulting scores plot (Figure 2A) shows clear separation between OM (blue) and RM (red) samples, with no overlap between the 95% confidence ellipses, confirming robust discrimination between the two mucus types.

The model's robustness and generalization were further supported by permutation and learning curve analyses. The permutation test (Figure 2B), based on 1000 random label shuffles, confirmed that none of the permuted models reached the observed AUC (*p* < 0.001), indicating that the discrimination was not due to chance. The learning curve analysis (Figure 2C) showed convergence between training and validation AUC values as sample size increased, confirming stable generalization and absence of overfitting. In contrast, alternative algorithms (XGBoost, Logistic Regression, Neural Networks) displayed diverging curves, with high training accuracy but unstable validation performance.

Finally, these results demonstrate that the optimized PLS‐DA model provides a statistically valid and biologically interpretable classification of OM and RM samples. The model achieved high, reproducible AUC values, showed no signs of overfitting, and significantly outperformed all random and alternative models. It was therefore selected for feature interpretation to identify the proteins driving the observed regional separation.

### 3.4 Functional Interpretation and GO‐Based Mapping

The optimized PLS‐DA model was used to identify the proteins that most strongly contributed to the discrimination between OM and RM mucus. Positive coefficients corresponded to proteins enriched in OM, whereas negative coefficients indicated higher abundance in RM (Figure S2). These discriminant proteins reflected coherent biological trends consistent with the enrichment analyses: OM was characterized by proteins involved in cytoskeletal organization, ciliary motility, and transport processes, whereas RM contained proteins related to metabolic regulation, protein synthesis, and immune and humoral defense. A list of most discriminant proteins and their biological functions is provided in Table S3.

To obtain a higher‐level functional overview, the discriminant proteins derived from the PLS‐DA model were functionally grouped according to their GO Biological Process annotations. GO terms with related molecular or cellular functions were manually merged into 29 predefined macro‐categories, providing a more interpretable and biologically meaningful representation (Table S4). Each protein was associated with one or several GO terms according to UniProtKB annotations, and the mean normalized absolute PLS‐DA coefficients within each macro‐category were used to quantify its relative functional contribution for OM and RM (Figure S4).

**FIGURE 3:**
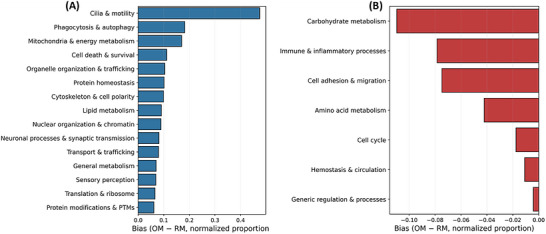
(A) Top biological macro‐categories enriched in the OM compared to the RM. Each bar represents the relative bias (OM–RM) calculated from the normalized mean absolute PLS‐DA coefficients within each macro‐category. Positive values indicate higher functional representation in the OM. (B) Top biological macro‐categories enriched in the RM compared to the OM. Each bar represents the relative bias (OM–RM) calculated from the normalized mean absolute PLS‐DA coefficients within each macro‐category. Negative values indicate higher functional representation in the RM.

In the OM, the most enriched macro‐categories were cilia and motility, phagocytosis and autophagy, mitochondria and energy metabolism, cell death and survival, and organelle organization and trafficking (Figure 3A). These functions highlight the dynamic and structural specialization of the olfactory mucosa, supporting neuronal turnover, vesicular transport, and sensory processing.

In contrast, the RM showed higher representation of macro‐categories related to carbohydrate metabolism, immune and inflammatory processes, cell adhesion and migration, amino acid metabolism, and hemostasis and circulation (Figure 3B). These functions reflect the metabolic and vascular specialization of the respiratory epithelium, ensuring mucosal maintenance, immune protection, and epithelial regeneration.

Overall, the GO‐based functional mapping integrates molecular discrimination patterns into a coherent biological framework. The agreement between the PLS‐DA functional contributions and the enrichment analyses confirms that the regional proteomic differences between OM and RM are not random but reflect distinct physiological roles within the nasal cavity; structural and sensory specialization in OM versus metabolic and defensive activity in RM.

### 3.5 Global and Macro‐Category–Based Reactome Enrichment Data Analysis

To gain an overview of the biological processes differentially represented at the pathway level in OM and RM, we performed a Reactome pathway enrichment analysis based on the proteins identified by LC–MS/MS and their PLS‐DA coefficients. The analysis was conducted both globally and within major functional macro‐categories defined from GO Biological Processes, linking molecular profiles to distinct physiological roles.

**FIGURE 4:**
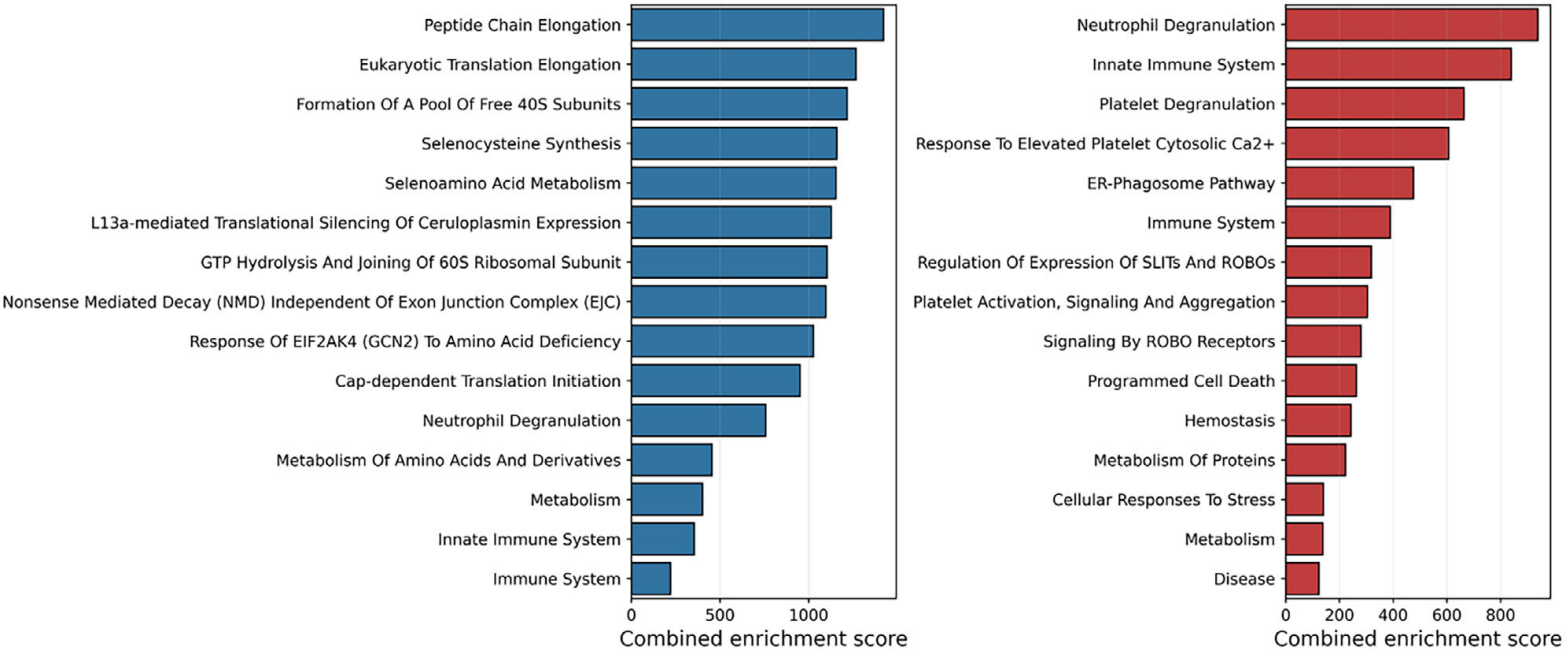
Reactome pathway enrichment analysis comparing OM and RM proteomes. The figure displays the top Reactome pathways significantly enriched in proteins contributing to the discrimination between OM (blue) and RM (red) according to the PLS‐DA model. Bars represent the combined enrichment scores calculated by Enrichr (Reactome 2022, Homo sapiens).

The global Reactome enrichment (Figure 4) revealed distinct functional profiles between the two mucus types. OM was mainly enriched in pathways related to translation, protein synthesis, and metabolism, including peptide chain elongation, ribosomal subunit assembly, and selenocysteine metabolism. RM showed predominant enrichment in innate immune and inflammatory pathways, such as neutrophil degranulation, platelet activation, and ER–phagosome interactions, reflecting its protective and immune‐specialized function.

Stratification by macro‐categories (Table 1 and Figures S4–S18) confirmed a clear functional polarization: OM was dominated by biosynthetic and metabolic activity, whereas RM was characterized by immune, hemostatic, and tissue‐repair processes. These findings emphasize the complementary specialization of the olfactory and respiratory mucosae in metabolic support versus immune protection.

**TABLE 1:** Reactome pathway enrichment within major functional macro‐categories differentiating olfactory (OM) and respiratory (RM) mucus proteomes.

Macro‐category	Dominant mucus type	Number of proteins identified (OM/RM)	Top pathways identified at OM according to the Reactome database	Top pathways identified at RM according to the Reactome database
Cilia & motility	OM ↑	34/13	Intraflagellar Transport; Cilium Assembly	Chylomicron Clearance; VLDL Clearance
Phagocytosis & autophagy	OM ↑	43/75	Scavenging by Class F Receptors; Chaperone‐Mediated Autophagy	CHL1 Interactions; MyD88 Deficiency (TLR2/4)
Mitochondria & energy metabolism	OM ↑	121/94	Pentose Phosphate Pathway; Citric Acid (TCA) Cycle and Respiratory Electron Transport	Mitochondrial Ca^2^^+^ Transport; ATP Synthesis by Chemiosmotic Coupling
Cell death & survival	OM ↑	192/202	Uptake and Function of Diphtheria Toxin; Release of Apoptotic Factors from Mitochondria	Metal Sequestration by Antimicrobial Proteins; Paradoxical Activation of RAF Signaling
Carbohydrate metabolism	RM ↑	27/27	Gluconeogenesis; Glucose Metabolism	Platelet Adhesion to Exposed Collagen; Gluconeogenesis
Immune & inflammatory processes	RM ↑	202/279	Scavenging by Class F Receptors; Neutrophil Degranulation	Activation of C3 and C5; Classical Antibody‐Mediated Complement Activation
Cell adhesion & migration	RM ↑	116/158	Uptake and Function of Diphtheria Toxin; Scavenging by Class F Receptors	Fibronectin Matrix Formation; MyD88 Deficiency (TLR2/4)
Amino acid metabolism	RM ↑	32/21	Cytosolic tRNA Aminoacylation; tRNA Aminoacylation	Cytosolic tRNA Aminoacylation; tRNA Aminoacylation

Summary of the key GO‐derived macro‐categories showing the strongest directional bias according to PLS‐DA coefficients. For each category, the dominant direction (OM or RM), the number of associated proteins, and the most significantly enriched Reactome pathways (Reactome 2022, Homo sapiens) are listed.

## 4 Discussion

The comparative proteomic and pathway‐level analyses revealed a clear functional dichotomy between OM and RM, reflecting that distinct physiologic processes of their respective epithelia are reflected in the overlying mucus [15, 25]. OM was dominated by pathways related to cilia formation, mitochondrial energy metabolism, and protein homeostasis, while RM was characterized by immune and inflammatory pathways, complement activation, hemostasis, and amino acid and carbohydrate metabolism. These results are consistent with previous studies describing the structural and metabolic specialization of the olfactory mucosa, which sustains (non‐motile) ciliary and mitochondrial activity for tissue renewal [56], essential for odorant detection and functioning of the olfactory system, and the immune and metabolic functions of the respiratory mucosa, highly significant for host defense [57, 58]. The convergence between LFQ, PLS‐DA modeling, and functional enrichment (GO and Reactome database) confirms that these regional differences are both statistically robust and biologically meaningful.

### 4.1 Metabolic Activity and Cellular Renewal

In OM, the enrichment of pathways such as intraciliary transport, respiratory electron transport, oxidative phosphorylation, and chaperone‐mediated autophagy indicates a metabolically active tissue optimized for ciliary regeneration and mitochondrial resilience. Processes linked to vesicular trafficking, cytoskeletal organization, and apoptotic regulation suggest a dynamic equilibrium between structural renewal and selective cell turnover, enabling sustained sensory function [59, 60, 61, 62].

The coexistence of autophagy, mitochondrial metabolism, and controlled apoptosis underlines the self‐maintaining nature of the olfactory epithelium, supporting both energy‐intensive receptor turnover and tissue protection from oxidative stress [60, 63, 64]. These findings position OM as a metabolically dynamic and structurally adaptive epithelium, specialized for continuous regeneration and sensory processing.

### 4.2 Immune Regulation and Mucosal Protection

In contrast, RM was enriched in immune and inflammatory pathways, including neutrophil degranulation, complement activation, and metal sequestration by antimicrobial proteins, as well as in cell adhesion and migration processes such as fibronectin matrix formation and MyD88‐dependent signaling. These pathways highlight the immune‐protective and structural barrier roles of the respiratory mucosa, enabling mucociliary clearance and tissue repair following environmental or microbial exposure [65, 66]. In addition, enrichment in carbohydrate and amino acid metabolism pathways reflects a coupling between energy supply and immune activity, characteristic of tissues engaged in constant defense and secretion [16, 67]. These results depict RM as a metabolically regulated immune barrier, maintaining homeostasis through coordinated inflammation, adhesion, and nutrient turnover.

### 4.3 Structural Specialization and Implications for Nasal Physiology

The complementary proteomic profiles of OM and RM underline two interdependent mucosal ecosystems within the nasal cavity. OM combines high mitochondrial activity, ciliary renewal, and autophagy to sustain sensory and structural resilience, whereas RM integrates immune signaling, adhesion, and metabolic regulation to ensure epithelial protection and clearance. This dual organization reflects an evolutionary division of labor: OM prioritizes structural and energetic regeneration, while RM provides immune and metabolic defense, maintaining the balance between sensory perception, protection, and mucosal homeostasis [68].

The distinct biochemical environments of OM and RM have major implications for nasal physiology and drug delivery. The energy‐rich, mitochondria‐active OM provides a suitable interface for neuroregeneration and potential nose‐to‐brain transport but may present barriers due to high oxidative and enzymatic activity. Conversely, the immune‐dominant RM favors mucociliary clearance and rapid secretion, which may limit drug residence time but could be exploited for formulations designed for mechanical transport or sustained local delivery [9, 69]. Understanding these regional molecular profiles can therefore guide the design of site‐specific intranasal therapies, balancing permeability, enzymatic stability, and immune compatibility [70].

Although this study offers the most comprehensive comparative proteomic characterization of OM and RM to date, certain limitations remain. The cohort size (*n* = 50) captures only part of the inter‐individual variability related to age, sex, or environment. Even if it is expected to find secreted factors or proteins of the extracellular matrix in these sample type, a substantial amount of the LC‐MS/MS identified proteins in this study are linked to GO‐terms that describe intra‐cellular processes and functions. This phenomenon might be caused by damaged tissue or released proteins during cell death as part of a tissue bleeding process. Also, it can´t be excluded those cells were sticking to the surface of the material that was used in the sample collection process. Moreover, the analysis was restricted to soluble proteins, while extracellular vesicles, lipids, and glycans may also shape mucus function. GO terms annotations could reflect multifunctional proteins rather than true pathway activity, highlighting a common limitation of proteomic enrichment analyses. Future studies combining proteomic, transcriptomic, and metabolomic data, as well as in vitro and ex vivo validation, are needed to link molecular signatures with epithelial physiology and drug permeability. Extending this integrative approach to patient cohorts and disease models will clarify how alterations in mucus composition reflect olfactory and respiratory health.

## 5 Conclusion

This study provides an integrated and quantitative characterization of the molecular specialization of human nasal mucus. Combining proteomics, machine learning, and pathway enrichment analyses, it reveals a distinct functional dichotomy between OM and RM. OM displays an energy‐intensive and biosynthetically active profile dominated by translation, mitochondrial metabolism, autophagy, and ciliary motility, consistent with its role as a neuroimmune interface supporting epithelial repair and sensory renewal. RM exhibits an immune‐active and metabolically regulated signature enriched in complement activation, antimicrobial defense, and hemostasis. These complementary systems demonstrate that mucus composition reflects the physiological specialization of their respective epithelia. Beyond their biological significance, these insights provide a molecular framework for targeted intranasal pharmacology, diagnostic biomarker discovery, and site‐specific drug delivery strategies.

## Author Contributions

R.T. performed data analysis and interpretation, and wrote the manuscript. A.K.H. and K.L. recruited participants and collected nasal mucus samples under the supervision of T.H., C.M., and F.R. contributed to supervision, funding acquisition and manuscript revision. P.H. and J.P. performed mass spectrometry measurements and data preprocessing. T.H. and K.S. were responsible for funding acquisition, conceived and supervised the overall project and critically reviewed and edited the manuscript. All authors discussed the results, revised the manuscript, and approved the final version.

## Conflicts of Interest

The authors declare no conflicts of interest.

## Supporting information




**Supporting File**: prca70049‐sup‐0001‐SuppMat.docx

## Data Availability

The data supporting the findings of this study are available from the corresponding author upon reasonable request. Due to privacy and ethical restrictions related to participant information, individual‐level metadata are not publicly available.
