# Supplementary material for: Distinct Functional Signatures of Human Olfactory and Respiratory Mucus Revealed by Proteomics Combined With Machine Learning
Source: Proteomics Clin Appl. 2026 Jul 17;20(4):e70049. doi: 10.1002/prca.70049 (PMC13377676; doi:10.1002/prca.70049)
Supplement: Supplementary file 1 — Supporting File: prca70049‐sup‐0001‐SuppMat.docx [file PRCA-20-e70049-s001.docx]

Supplementary of

Distinct Functional Signatures of Human Olfactory and Respiratory Mucus Revealed by Proteomics combined with Machine Learning

Romain Topalian^a,b+*^, Anna Kristina Hernandez^c,d,e+^, Karoline Lantzsch^c^, Philipp Hubel^f^, Chrystelle Mavoungou^b^, Frank Rosenau^a^, Jens Pfannstiel^f^, Thomas Hummel^c^, Katharina Schindowski^b*^

^a^Institute of Pharmaceutical Biotechnology, Ulm University, Albert-Einstein-Allee 11, 89081 Ulm, Germany

^b^Institute for Applied Biotechnology, Biberach University of Applied Sciences, Karlstraße 6-11, 88400 Biberach, Germany

^c^Smell & Taste Clinic, Department of Otorhinolaryngology, Faculty of Medicine Carl Gustav Carus, Technische Universität Dresden, Fetscherstrasse 74, 01307 Dresden, Germany

^d^Department of Otolaryngology – Head and Neck Surgery, Philippine General Hospital, University of the Philippines – Manila, Taft Avenue, 1000 Manila, Philippines

^e^Department of Otolaryngology – Head and Neck Surgery, Asian Hospital and Medical Center, 2205 Civic Drive, Filinvest Corporate City, 1780 Muntinlupa, Philippines

^f^ Core Facility Hohenheim, Mass Spectrometry Unit, University of Hohenheim, Stuttgart, Germany

^*^Corresponding authors

^+^Contributed equally

Email: [romain.topalian@uni-ulm.de](mailto:romain.topalian@uni-ulm.de); schindowski@hochschule-bc.de

Table S1: Comparison of imputation methods applied to the LFQ proteomics dataset from OM and RM samples. Twelve imputation strategies were evaluated to reconstruct missing protein intensity values and minimize bias in downstream analyses. Each method was assessed based on Root Mean Square Error (RMSE) and Mean Absolute Error (MAE) calculated between original and reconstructed data after artificial masking of known values.

| **Method** | **RMSE** | **MAE** |
| --- | --- | --- |
| SVD-like Imputation | 0.872 | 0.622 |
| Iterative Imputation | 0.880 | 0.616 |
| PCA Imputation | 0.897 | 0.638 |
| KNN Imputation | 0.987 | 0.695 |
| Mean Imputation | 1.508 | 1.163 |
| DAE Imputation | 1.566 | 1.181 |
| BPCA-like Imputation | 1.587 | 1.206 |
| Multivariate Normal (mean by column) | 1.594 | 1.208 |
| AutoEncoder Imputation | 1.659 | 1.272 |
| QRILC-like Imputation | 3.207 | 2.686 |
| MinDet Imputation | 6.385 | 5.994 |
| VAE Imputation | 1.14 × 10⁴ | 8.19 × 10³ |
| EM Imputation | 5.28 × 10³² | 1.92 × 10³¹ |


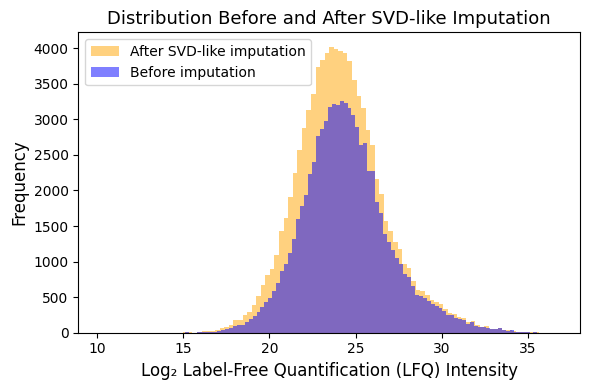


Figure S1: Histogram comparison of log₂-transformed Label-Free Quantification (LFQ) protein intensities before (blue) and after (orange) SVD-like imputation. Each curve represents the frequency distribution of all quantified protein intensity values across the dataset. The “before” histogram includes only the experimentally observed values, whereas the “after” histogram also contains values reconstructed for missing data points using a Singular Value Decomposition (SVD)–based method.

Table S2: Results of the 1D Gene Ontology (GO) enrichment analysis comparing nasal OM and RM. Each row represents a significantly enriched GO Slim term (FDR < 0.05) identified using Welch’s test statistics on normalized LFQ data. “Type” indicates the GO category (GOBP: Biological Process, GOCC: Cellular Component), “Score” corresponds to the Welch’s difference between OM and RM (positive values = enrichment in OM; negative values = enrichment in RM), and “FDR” represents the Benjamini–Hochberg–corrected significance value.

| **Type** | **Name** | **Size** | **Score** | **Benj. Hoch. FDR** | **Mean** |
| --- | --- | --- | --- | --- | --- |
| GOCC slim name | immunoglobulin complex | 88 | -0.80799 | 1.83E-31 | -0.74824 |
| GOCC slim name | cilium | 115 | 0.5988 | 3.99E-21 | 0.86582 |
| GOBP slim name | microtubule-based process | 198 | 0.41132 | 4.00E-15 | 0.6039 |
| GOCC slim name | microtubule | 129 | 0.47854 | 7.83E-14 | 0.714385 |
| GOBP slim name | microtubule-based movement | 120 | 0.4826 | 2.85E-12 | 0.69372 |
| GOCC slim name | microtubule organizing center | 163 | 0.39764 | 2.32E-11 | 0.589387 |
| GOCC slim name | axoneme | 45 | 0.65271 | 8.51E-08 | 0.984761 |
| GOBP slim name | killing of cells of another organism | 32 | -0.76004 | 5.88E-07 | -0.90494 |
| GOBP slim name | cilium organization | 75 | 0.47764 | 6.21E-06 | 0.708144 |
| GOBP slim name | cell killing | 50 | -0.5478 | 8.95E-05 | -0.58948 |
| GOCC slim name | centrosome | 117 | 0.34738 | 2.29E-04 | 0.550991 |
| GOBP slim name | microtubule cytoskeleton organization | 95 | 0.33106 | 7.26E-02 | 0.515855 |
| GOBP slim name | cytoskeleton-dependent intracellular transport | 71 | 0.36444 | 2.62E-01 | 0.551331 |
| GOBP slim name | zymogen activation | 20 | -0.60746 | 4.41E+00 | -0.64448 |
| GOBP slim name | tRNA aminoacylation for protein translation | 27 | 0.49656 | 0.000130713 | 0.66228 |
| GOBP slim name | tRNA metabolic process | 44 | 0.38082 | 0.000200395 | 0.556964 |
| GOBP slim name | cellular aldehyde metabolic process | 34 | 0.41194 | 0.000462966 | 0.635788 |
| GOBP slim name | pattern specification process | 37 | 0.3588 | 0.00195269 | 0.585984 |
| GOBP slim name | synapse pruning | 5 | -0.95565 | 0.00236082 | -1.4123 |
| GOBP slim name | mitotic cell cycle | 29 | 0.39029 | 0.00279974 | 0.572879 |


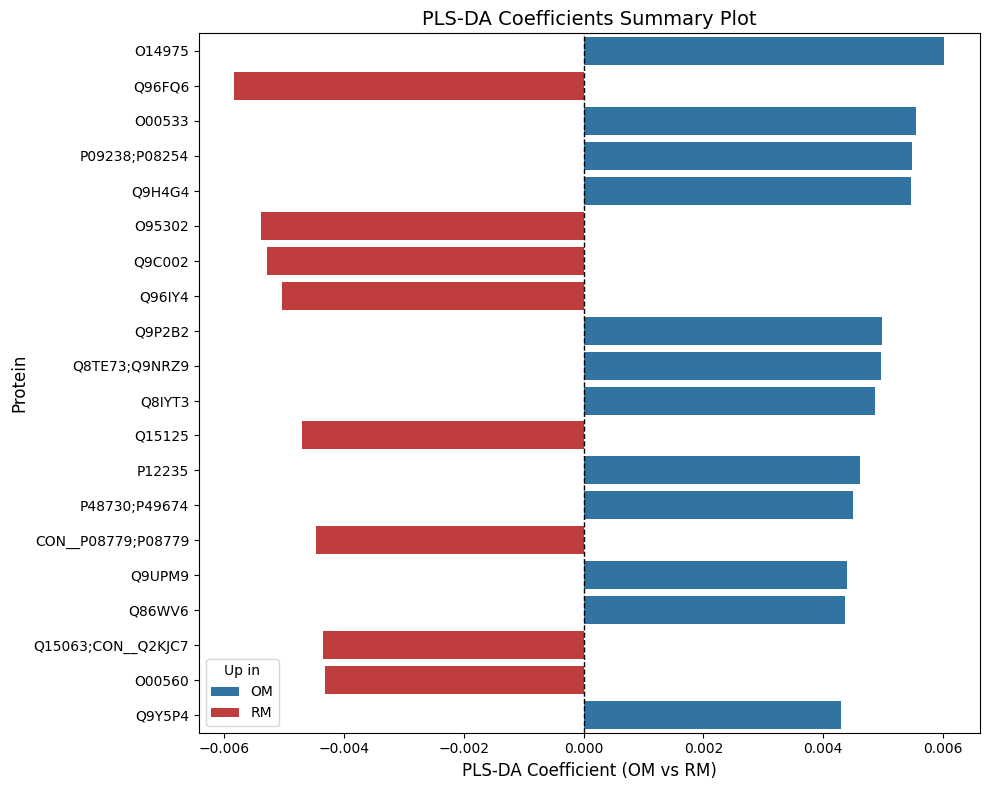


Figure S2: Summary plot of the top discriminant proteins identified by the optimized PLS-DA model. Each bar represents the PLS-DA coefficient associated with a given protein, indicating its contribution to the discrimination between OM and RM. Positive coefficients (blue) correspond to proteins more abundant in OM, while negative coefficients (red) correspond to proteins enriched in RM. Only the 15 most influential proteins are displayed for clarity.

Table S3: List of the most discriminant proteins identified by the optimized PLS-DA model differentiating OM and RM samples. Each protein is reported with its UniProt identifier, the mucus type in which it is predominantly expressed, and its main biological function inferred from literature and Gene Ontology annotation.

| **Protein ID** | **Protein Name (main known function)** | **Associated mucus** | **Biological function / process** |
| --- | --- | --- | --- |
| O14975 | Long-chain fatty acid transport protein 2 | OM | Fatty acid metabolism, lipid transport, mitochondrial β-oxidation |
| Q96FQ6 | Protein S100-A16 | RM | Calcium-binding protein involved in epithelial differentiation and metabolic regulation |
| O00533 | Neural cell adhesion molecule L1-like protein | OM | Cell adhesion, axon guidance, neuronal connectivity |
| P09238; P08254 | Stromelysin-2 / Stromelysin-1 (MMP family) | OM | Extracellular matrix remodeling, tissue repair, proteolysis |
| Q9H4G4 | Golgi-associated plant pathogenesis-related protein 1 | OM | Golgi trafficking, vesicle transport, secretion processes |
| O95302 | Peptidyl-prolyl cis-trans isomerase FKBP9 | RM | Protein folding in endoplasmic reticulum, stress response |
| Q9C002 | Normal mucosa of esophagus-specific gene 1 protein | RM | Cell differentiation and epithelial homeostasis |
| Q96IY4 | Carboxypeptidase B2 | RM | Regulation of fibrinolysis and proteolytic activity |
| Q9P2B2 | Prostaglandin F2 receptor negative regulator | OM | Modulation of prostaglandin signaling and inflammation |
| Q8TE73; Q9NRZ9 | Dynein axonemal heavy/intermediate chains | OM | Ciliary motility and axonemal transport |
| Q8IYT3 | Coiled-coil domain-containing protein 170 | OM | Cytoskeletal organization and cell polarity |
| Q15125 | 3-β-Hydroxysteroid-Δ(8),Δ(7)-isomerase | RM | Steroid metabolism, lipid biosynthesis |
| P12235 | ADP/ATP translocase 1 | OM | Mitochondrial energy metabolism, ATP/ADP exchange |
| P48730; P49674 | Casein kinase I δ / ε | OM | Phosphorylation signaling, circadian rhythm regulation |
| P08779 | Keratin 16 | RM | Structural component of epithelial cells, cytoskeleton integrity |
| Q9UPM9 | B9 domain-containing protein 1 | OM | Ciliogenesis and ciliary signaling |
| Q86WV6 | Stimulator of interferon genes (STING) protein | OM | Innate immune signaling and antiviral response |
| Q15063; Q2KJC7 | Periostin | RM | Extracellular matrix organization, cell adhesion, wound healing |
| O00560 | Syntenin-1 | RM | Membrane trafficking, exosome biogenesis, cell signaling |
| Q9Y5P4 | Ceramide transfer protein | OM | Lipid transport and sphingolipid homeostasis |

Table S4: Distribution of proteins across the 29 manually defined biological macro-categories derived from GO Biological Process annotations. Each macro-category was generated by merging functionally related GO terms.

| **MacroCategory** | **Number of Proteins** |
| --- | --- |
| Generic regulation & processes | 2697 |
| Other | 2318 |
| General metabolism | 2049 |
| Organelle organization & trafficking | 1419 |
| Development & morphogenesis | 1337 |
| Stress response | 1316 |
| Transport & trafficking | 1152 |
| Signal transduction | 1041 |
| Protein modifications & PTMs | 979 |
| Cytoskeleton & cell polarity | 714 |
| Immune & inflammatory processes | 703 |
| Cell death & survival | 611 |
| Nuclear organization & chromatin | 481 |
| Transcriptional regulation | 444 |
| Cell adhesion & migration | 415 |
| Homeostasis & balance | 397 |
| Neuronal processes & synaptic transmission | 341 |
| Mitochondria & energy metabolism | 334 |
| Cell cycle | 305 |
| Translation & ribosome | 288 |
| Lipid metabolism | 272 |
| RNA metabolism & splicing | 221 |
| Phagocytosis & autophagy | 193 |
| Cilia & motility | 115 |
| Protein homeostasis | 107 |
| Hemostasis & circulation | 91 |
| Sensory perception | 78 |
| Carbohydrate metabolism | 76 |
| Amino acid metabolism | 76 |
| Genome stability & transposable elements | 1 |


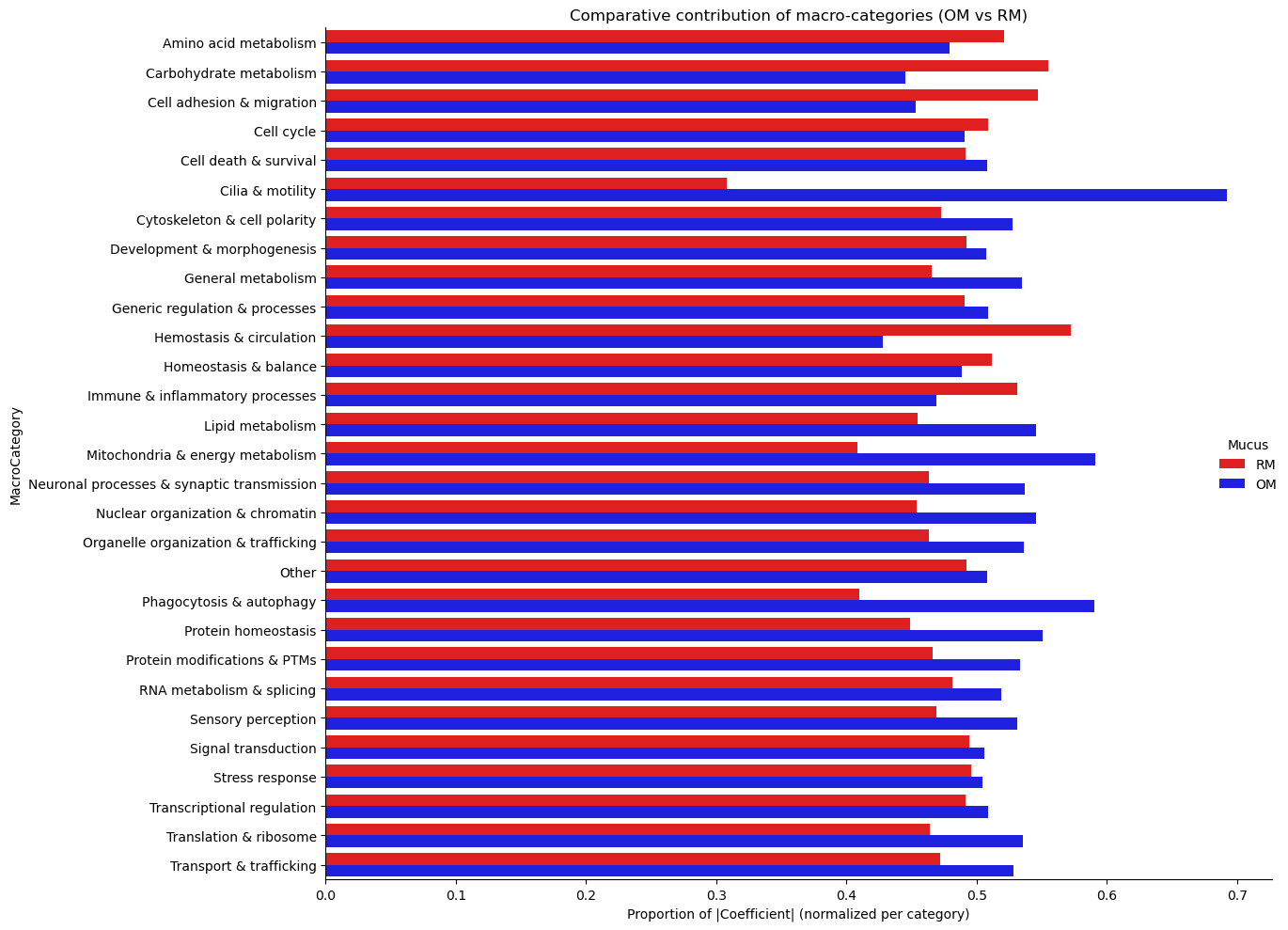


Figure S3: Comparative contribution of all biological macro-categories to the discriminant model for olfactory (OE mucus, blue) and respiratory (RE mucus, red) samples. Each bar represents the normalized proportion of the absolute PLS-DA coefficients within each macro-category. The figure illustrates the relative weight of each functional group in the overall discrimination model.


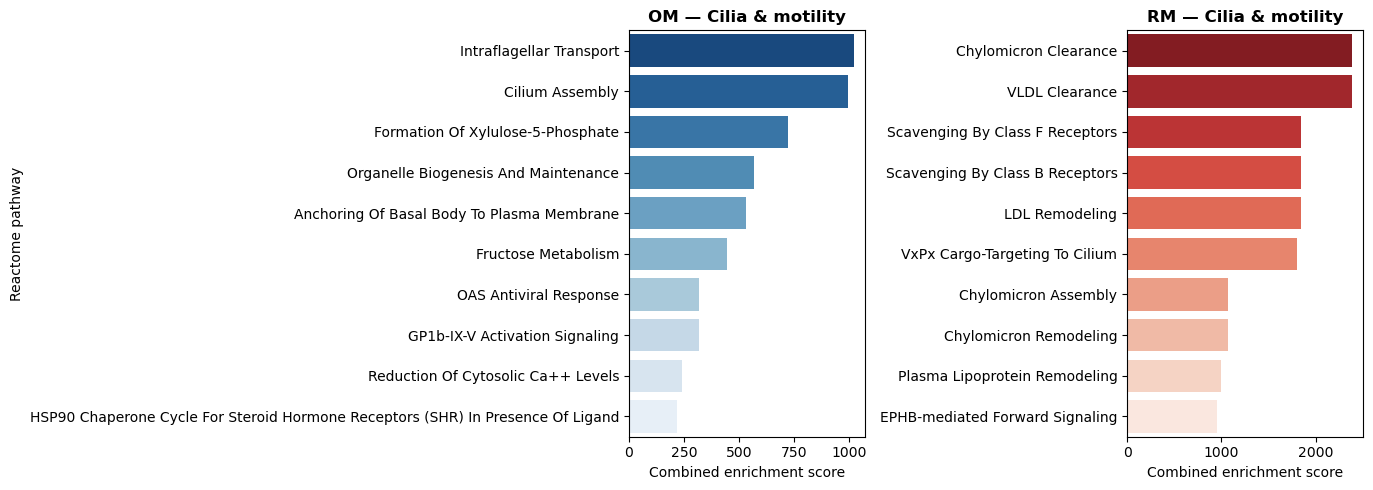

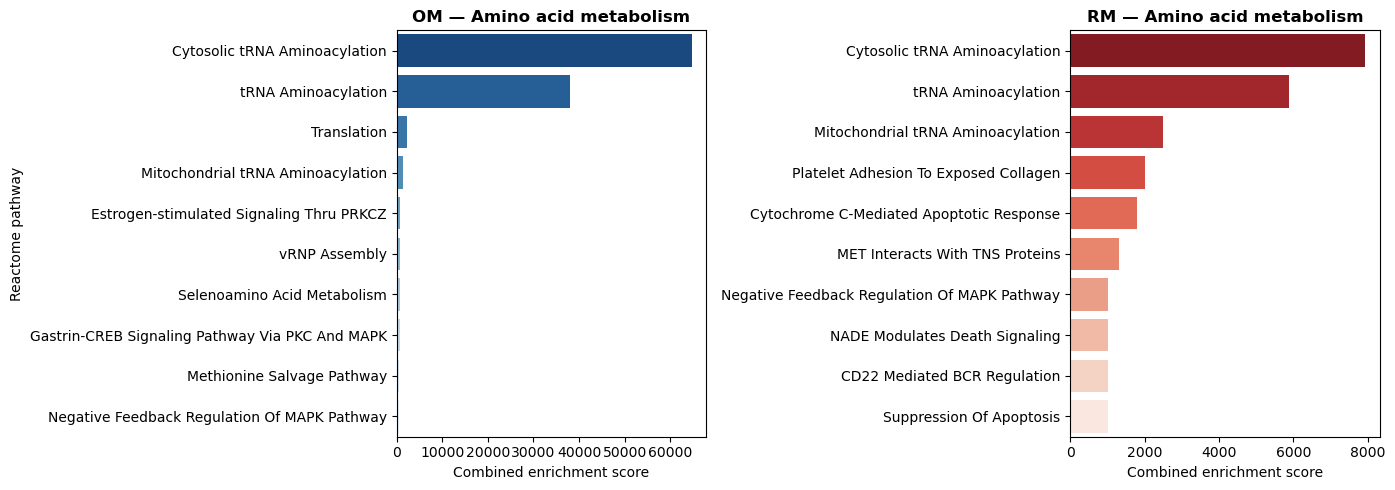


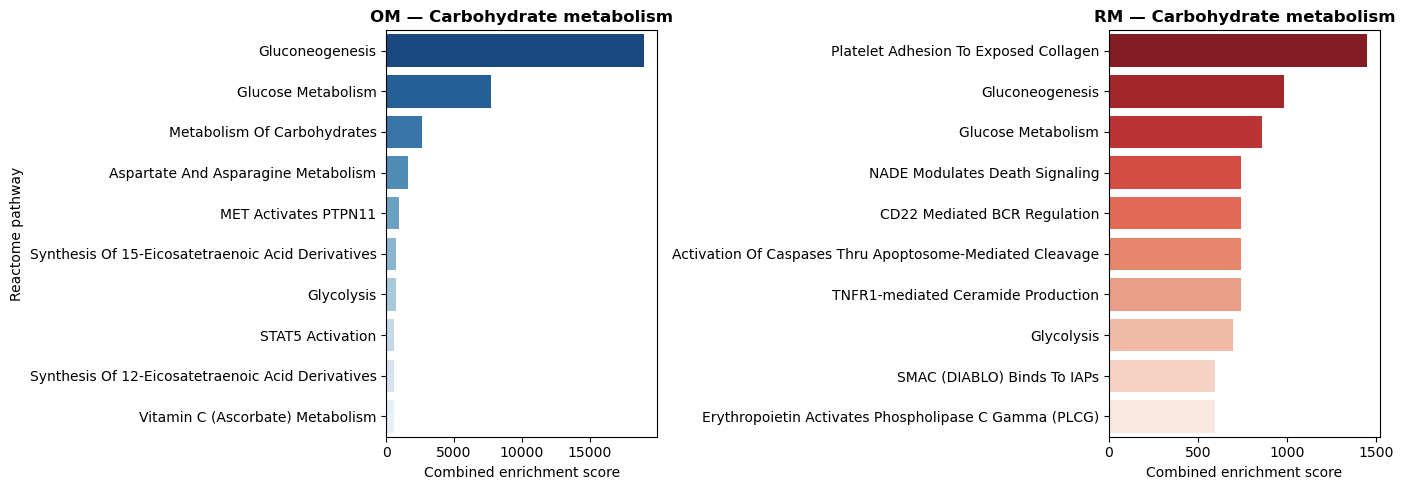

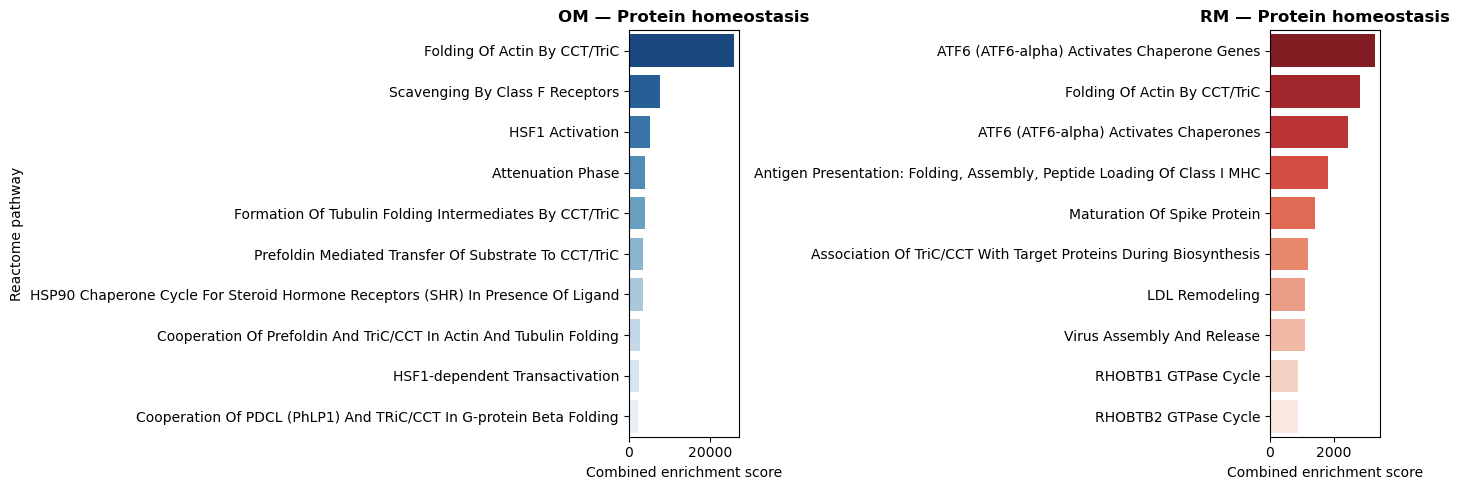

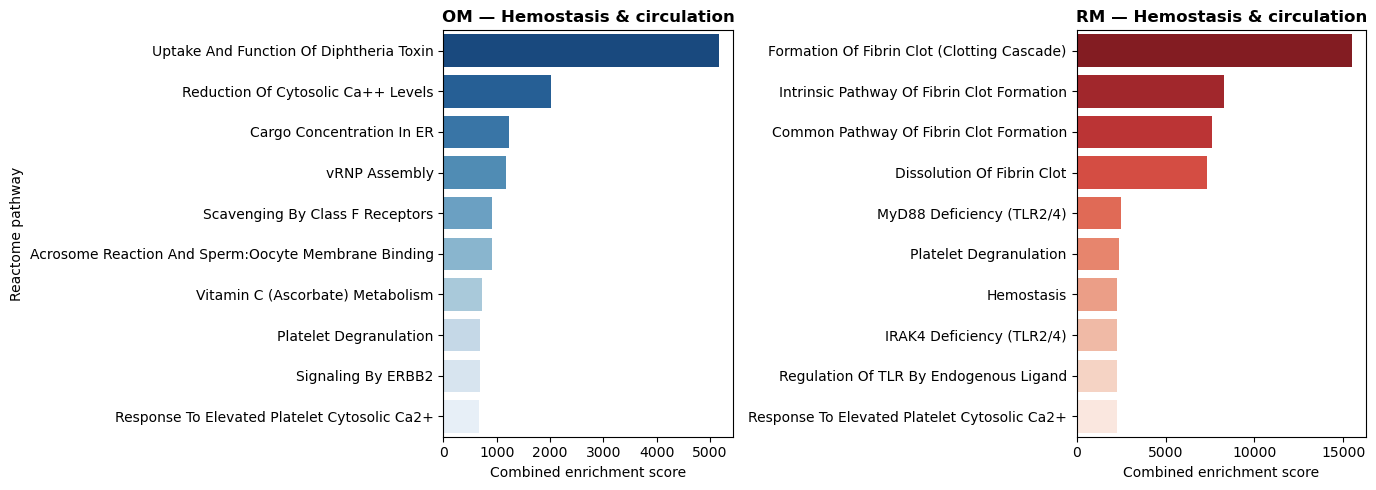

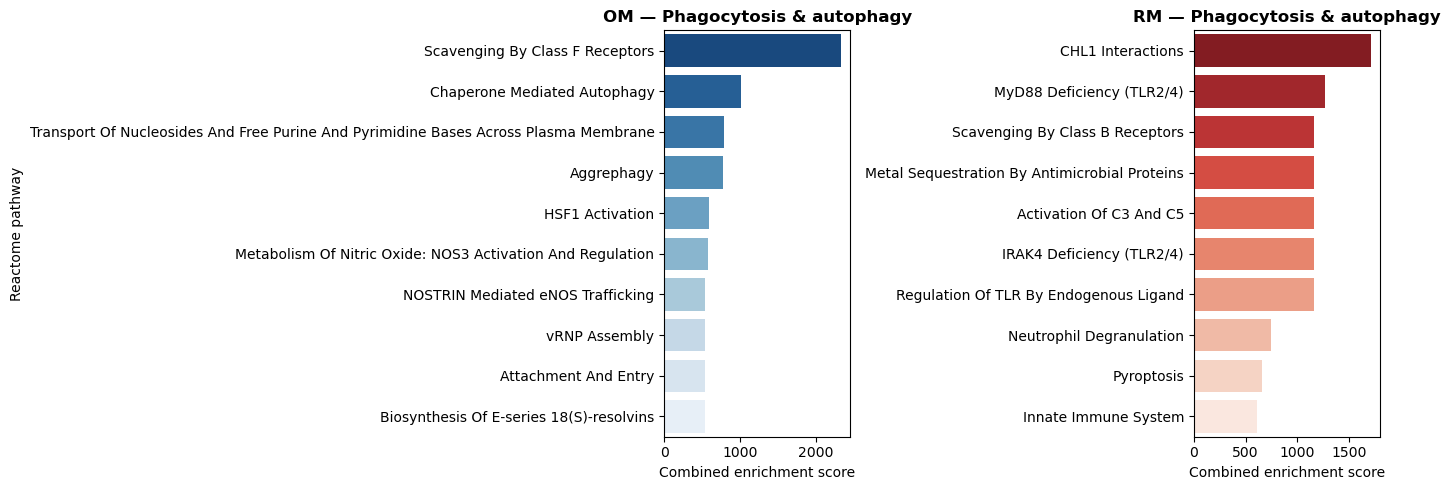

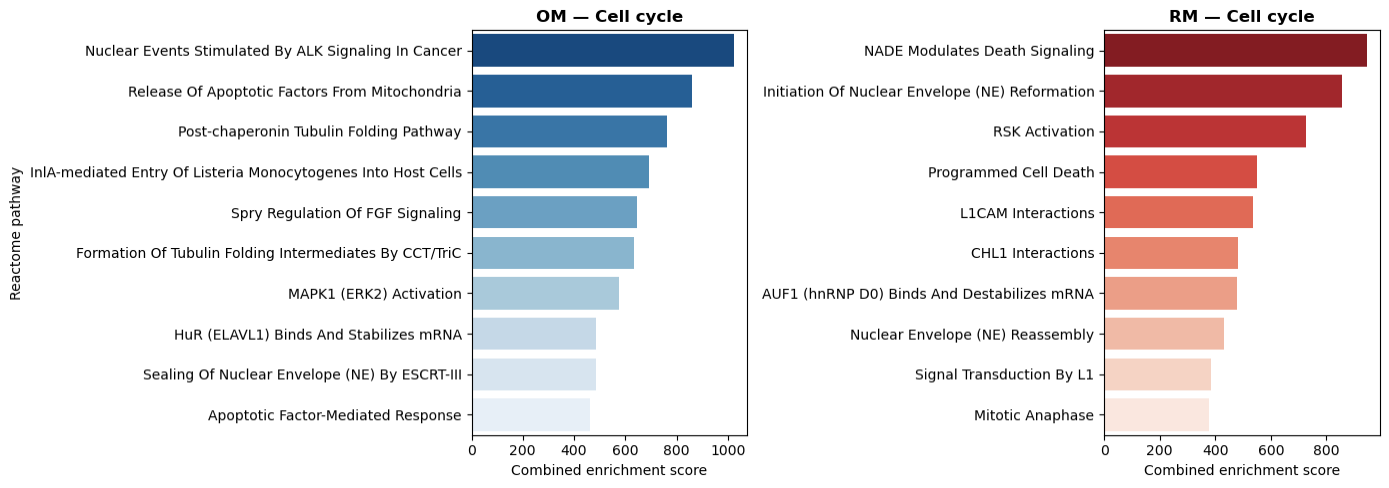

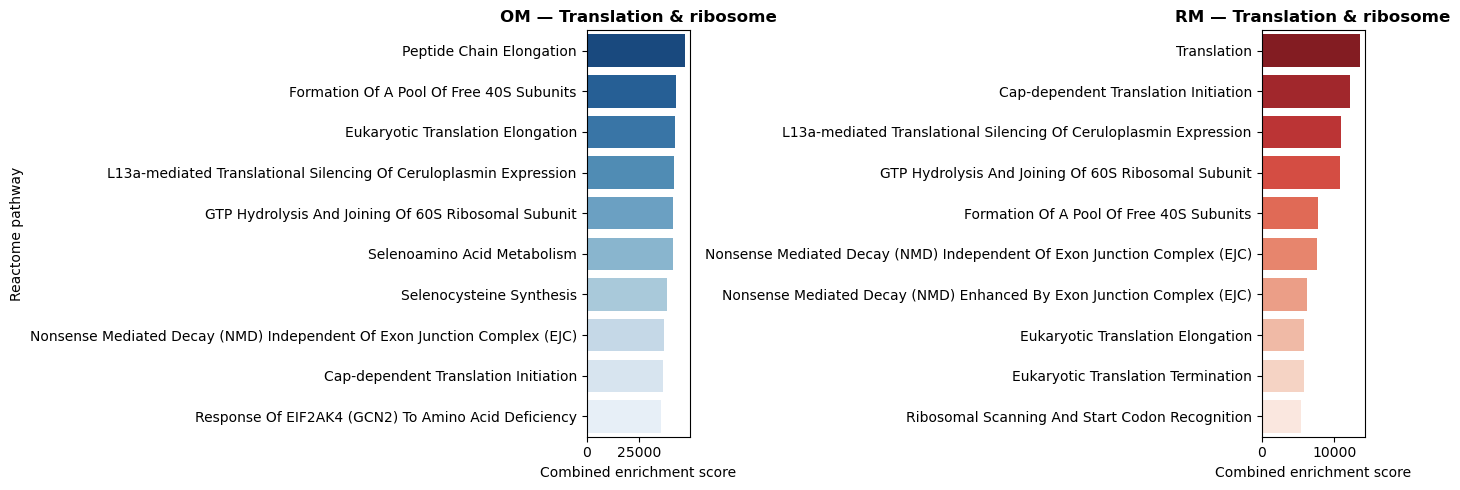

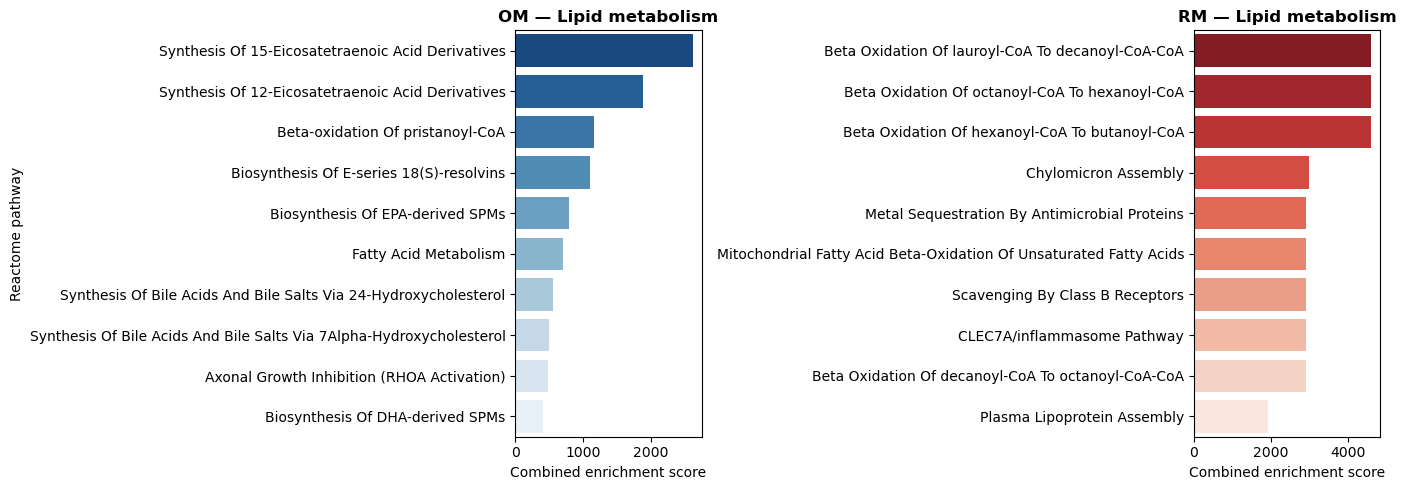

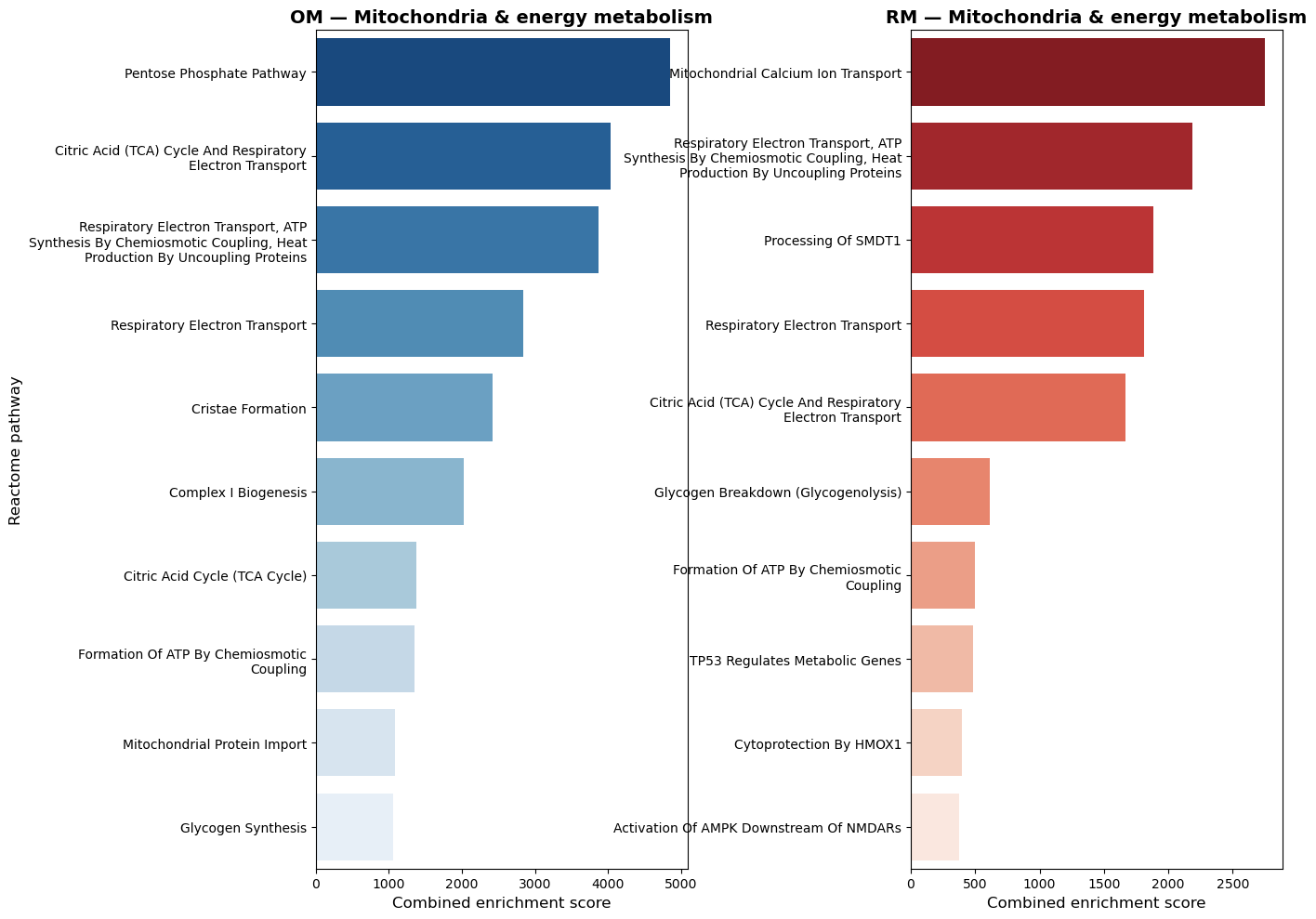

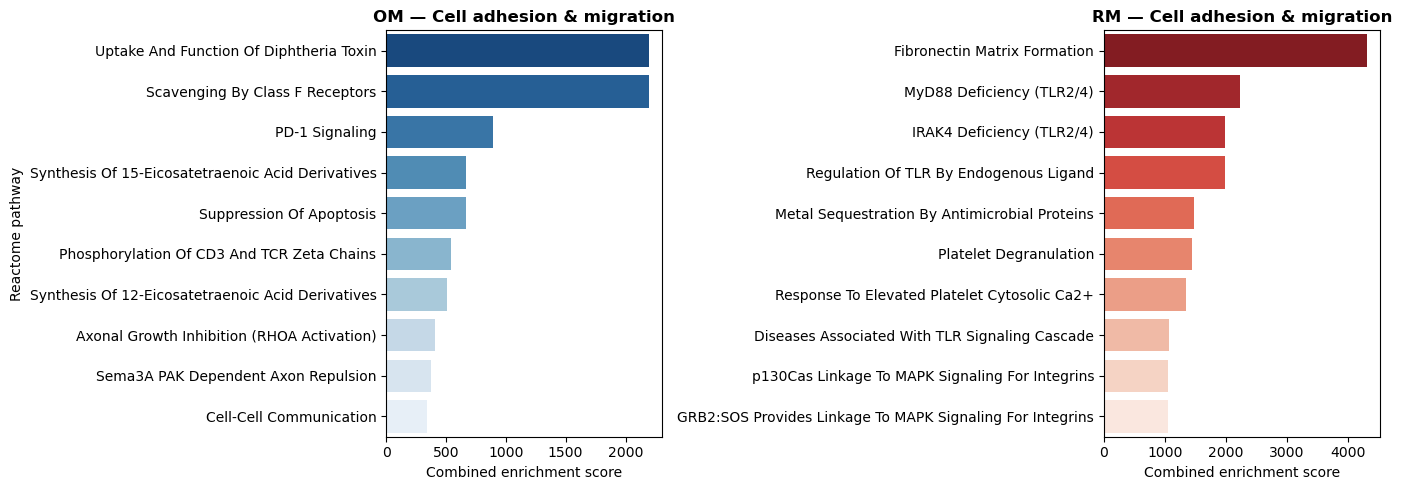

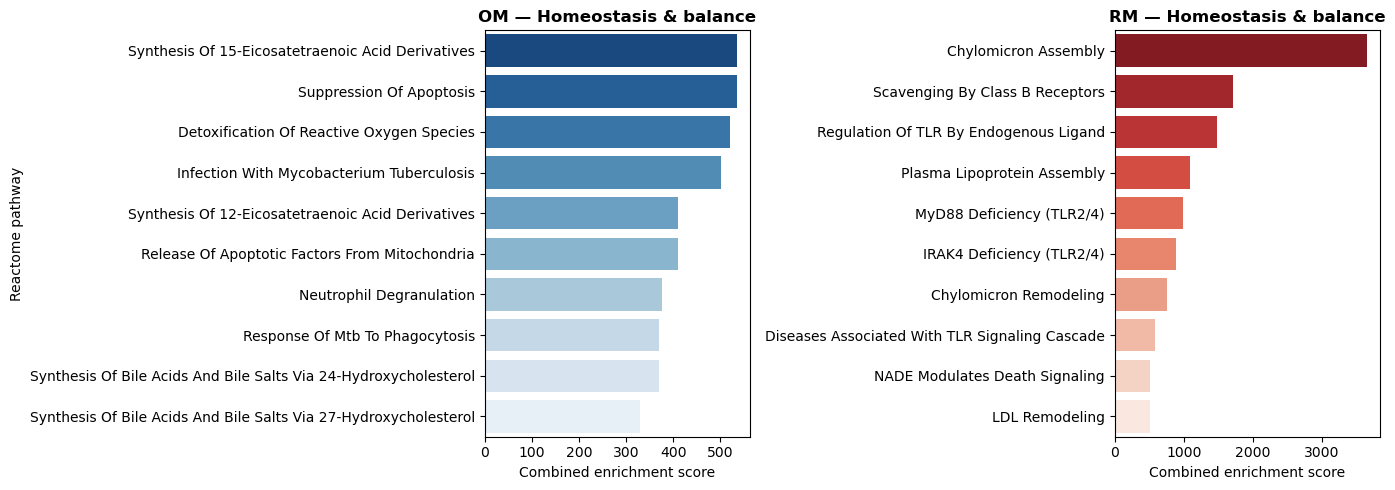

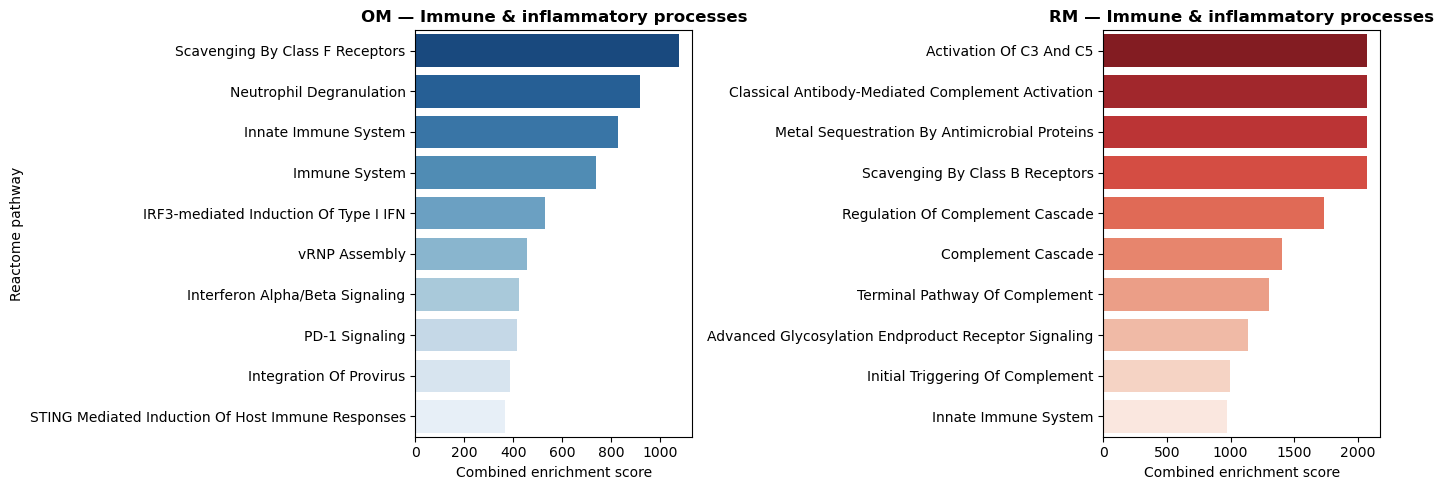

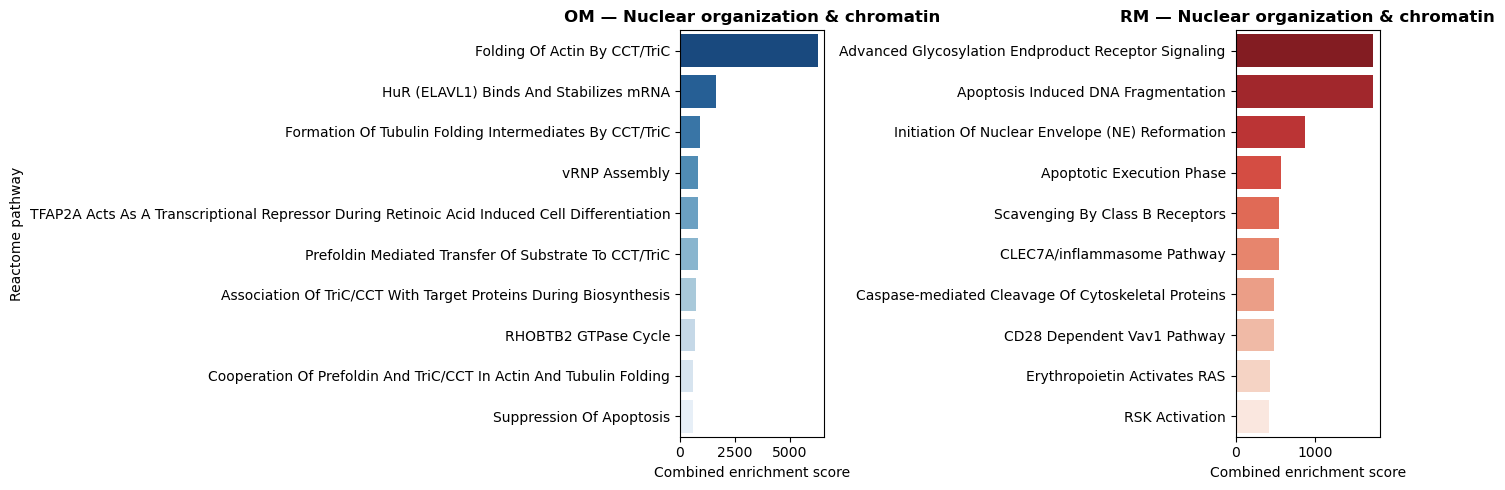


Figure S4-S18: **Reactome pathway enrichment within major GO-derived macro-categories for OM and RM.** Each panel shows the top Reactome pathways enriched in the corresponding macro-category.
